# Improved Biological Control of Bacterial Leaf Blight Using a Surfactant Complex of CO_2_ Micro-Nanobubbles Coated with Crude Ethyl Acetate Extract of *Trichoderma polyalthiae* UBZSN2-1

**DOI:** 10.3390/plants15020245

**Published:** 2026-01-13

**Authors:** Wasan Seemakram, Thanapat Suebrasri, Saranya Chantawong, Sornamol Traiphop, Sriprajak Krongsuk, Jirawat Sanitchon, Thanawan Gateta, Sophon Boonlue

**Affiliations:** 1 Department of Microbiology and Parasitology, Faculty of Medical Science, Naresuan University, Phitsanulok 65000, Thailand; wasans@nu.ac.th; 2 Division of Basic and Preclinical Sciences, Institute of Science and General Education, Nakhon Ratchasima College, Nakhon Ratchasima 30000, Thailand; s.thanapat@nmc.ac.th; 3 School of Preclinical Sciences, Institute of Science, Suranaree University of Technology, Nakhon Ratchasima 30000, Thailand; saranya.ch@sut.ac.th; 4 Institute of Nanomaterials Research and Innovation for Energy (IN-RIE), Khon Kaen University, Khon Kaen 40002, Thailand; so.traiphop@gmail.com (S.T.); srikro@kku.ac.th (S.K.); 5 Department of Physics, Faculty of Science, Khon Kaen University, Khon Kaen 40002, Thailand; 6 Department of Agronomy, Faculty of Agriculture, Khon Kaen University, Khon Kaen 40002, Thailand; jirawat@kku.ac.th; 7 Department of Microbiology, Faculty of Science, Khon Kaen University, Khon Kaen 40002, Thailand; thanawan.gateta@kkumail.com

**Keywords:** bioactive compound, carbon dioxide nanobubbles, endophytic fungi, surfactants

## Abstract

The bacterium *Xanthomonas oryzae* pv. *oryzae* is an important pathogen that causes wilt leaf blight disease in rice (*Oryza sativa* L.), leading to a reduction in rice yield. Therefore, this study aimed to investigate the potential of a surfactant complex composed of CO_2_ nanobubbles (CO_2_-NBs) coated with sorbitan monostearate (Sp60) and a crude extract of *Trichoderma polyalthiae* as active ingredient delivery agents for controlling leaf blight under both laboratory and greenhouse conditions. The addition of Sp60 and crude extract as surfactants significantly influenced the size uniformity and stability of CO_2_-NBs at the nano level, with the nanobubbles remaining intact in water for up to 14 days. In addition, CO_2_-NBs with crude extract and Sp60 reduced the severity of wilt, with an minimum inhibitory concentration (MIC) value of 64 µg/mL and an minimum bactericidal concentration (MBC) value of 128 µg/mL, and inhibited the disease by more than 50% in greenhouse conditions. Therefore, this study presents a creative and eco-friendly approach to managing bacterial leaf blight in rice that is innovative and relevant to sustainable plant protection.

## 1. Introduction

Rice (*Oryza sativa* L.) is one of the most important staple food crops in several countries, especially Thailand, in terms of consumption and agricultural export. According to agricultural statistics, rice cultivation covers 8.67 million hectares, with a combined yield of 27.01 million tons [1]. Currently, rice cultivation faces major problems, including bacterial leaf blight. Under the influence of the monsoon season, Thailand enters a 6-month rainy period, resulting in a tropical climate with an average temperature between 18 and 37 °C. This creates an optimal environment for disease outbreaks, especially bacterial leaf blight, which is caused by *Xanthomonas oryzae* pv. *oryzae* [2]. This has led to widespread and severe outbreaks, making it difficult to control. Bacterial leaf blight can reduce the quality and yield of rice worldwide [3].

Several chemicals, such as zinc thiazole, copper hydroxide, copper oxychloride, and tribasic copper sulfate [4], have been used to control bacterial blight in rice caused by *X. oryzae*. The application of these chemicals must be approached with caution, as improper use or excessive quantities can lead to adverse effects on both the environment and the health of consumers and farmers [5]. Currently, biocontrol is becoming increasingly popular because of its safety for consumers and its environmentally friendly nature [6]. However, the use of microorganisms is limited due to their unstable efficacy, and the efficiency also depends on the environment. Biocontrol using microorganisms or their metabolites has emerged as a safer and sustainable alternative. Endophytic fungi, in particular, produce a variety of bioactive compounds that can directly inhibit pathogen growth, interfere with biofilm formation, and induce systemic resistance in rice [7]. Currently, nanobubble technology has been increasingly applied in agriculture due to the unique physicochemical properties of the nanobubbles it produces. Unlike conventional gas–liquid systems, nanobubbles possess high stability, large surface-area-to-volume ratios, and improved gas transfer efficiency, enabling them to remain suspended in solution for extended periods [1,2]. In agricultural applications, these characteristics allow nanobubbles to enhance oxygen or CO_2_ availability in plant tissues, stimulate photosynthetic activity, promote nutrient uptake, and improve rhizosphere microbial activity [5]. Furthermore, when used as carriers, nanobubbles can deliver bioactive compounds more efficiently to plant tissues due to their nanoscale size, which enables penetration through stomata and intercellular spaces. This leads to better adhesion on leaf surfaces, improved uptake of active compounds, and more consistent biological performance compared with conventional liquid formulations [7].

Hence, a change to using a bioactive compound from endophytic fungi instead of fungicides and microorganisms can control plant pathogens. It is an additional option to solve the disadvantages of the aforementioned microorganisms and chemicals [8]. Typically, CO_2_ from the atmosphere diffuses into the substomatal cavities of plant leaves and then transfers to carboxylation sites [9]. Traiphop et al. [10] reported that using CO_2_ nanobubbles (CO_2_-NBs) can enhance the growth of rice through increased photosynthesis rates. Nanobubbles (NBs) are generated when gas is introduced into a liquid under high shear or pressure conditions followed by rapid decompression, leading to the formation of bubbles with diameters below 1000 nm [11,12]. These bubbles are stabilized primarily by electrostatic repulsion at the gas–liquid interface, due to the presence of surface charges preventing their coalescence [13,14,15]. Additionally, surfactants or natural organic molecules can enhance the stability of NBs by forming a protective interfacial layer. In this study, CO_2_ nanobubbles (CO_2_-NBs) were produced using a pressurized gas dispersion method, and the resulting bubbles, typically 100–700 nm in diameter, served as carriers to deliver bioactive compounds efficiently to plant tissues.

Our previous reports showed that a new species of endophytic fungi, *Trichoderma polyalthiae* UBZSN2-1 was isolated from stems of *Polyalthia debilis* in Ubon Ratchatani Province, Thailand [16]. a crude extract of *Trichoderma polyalthiae* UBZSN2-1 exhibited strong antimicrobial activity against Gram-positive and Gram-negative bacteria [17]. In addition, CO_2_ nanobubbles (CO_2_-NBs) can enhance the photosynthesis rates and growth of rice [10]. Nevertheless, there is still a knowledge gap regarding the applications of both bioactive compounds and CO_2_-NBs, especially in terms of their interoperability. Therefore, the application of nanobubbles in this study aims to exploit their enhanced delivery efficiency to improve the stability and disease-control effectiveness of fungal bioactive compounds to control bacterial leaf blight in rice. The results obtained in this work position this method as an innovative and environmentally friendly approach for managing bacterial leaf blight in rice or leaf diseases in other plants, contributing to sustainable plant protection.

## 2. Results

### 2.1. Screening of Bioactive Compounds from T. polyalthiae UBZSN2-1

**Figure 1:**
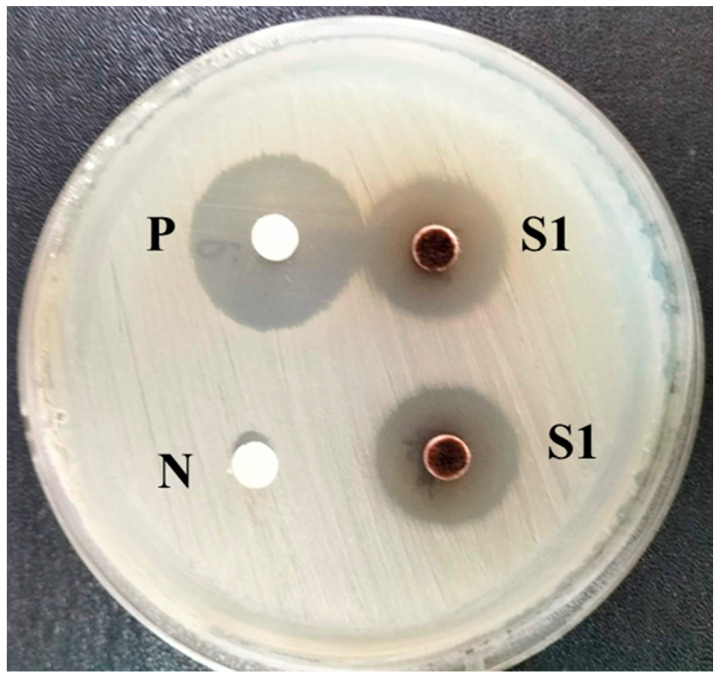
The antagonistic activity of crude ethyl acetate extract of *T. polyalthiae* UBZSN2-1 against *Xanthomonas oryzae* pv. *oryzae*. (P, positive; N, negative and S1, crude ethyl acetate extract).

The antibacterial activity of the crude extract was assessed using the disc diffusion technique (Figure 1). Notably, the crude fungal extract at a concentration of 125 µg/mL exhibited clear inhibitory activity against *X. oryzae* pv. *oryzae*, could produce a clear zone around the agar plug at 20 mm and a positive control at 22 mm. The diameter of the inhibition zone produced by the extract was comparable to that approximate by the antibiotic control, indicating that the crude extract possesses potent antibacterial properties.

### 2.2. Size Distribution and Polydispersity Index (PDI)

**Figure 2:**
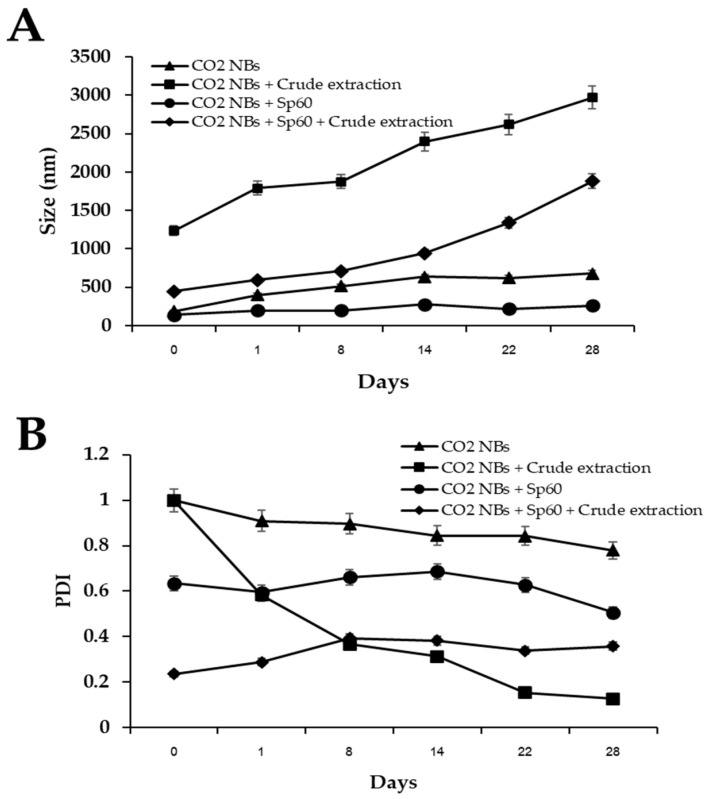
Average sizes of CO_2_-NBs for different formulations (**A**) and polydispersity index (PDI) (**B**).

The average size of CO_2_-NBs under various conditions is shown in Figure 2A. The size of CO_2_ NBs coated with crude extract from *T. polyalthiae* UBZSN2-1 and an Sp60 surfactant at days 0 to 28 was examined. The treatment formulations using the Sp60 surfactant (CO_2_ NBs + Sp60) (comparison treatment) had bubble sizes of 140 to 259 nm, and the treatment formulations using CO_2_ NBs + Sp60 + crude extract had bubble sizes of 446 to 1881 nm. In contrast, the treatment without Sp60 resulted in bubble sizes between 187 and 680 nm for CO_2_ NBs and between 1235 and 2972 nm for CO_2_ NBs plus crude extract.

The polydispersity index (PDI) of nanobubbles in different formulations indicates the degree of uniformity in particle size distribution (Figure 2B). CO_2_-NBs without any surfactant exhibited a PDI value around 1.0–0.7, indicating a polydisperse particle size distribution. In contrast, adding the crude extract alone resulted in polydisperse distributions, with a PDI value of 1.0 on day 0 and a PDI value ranging from 0.5 to 0.12 on days 1–28, indicating a monodisperse particle size distribution. The CO_2_-NBs + Sp60 treatment resulted in PDI values of around 0.5–0.6, suggesting some variability in particle size. Interestingly, when CO_2_-NBs + Sp60 were combined with crude extract, the PDI generally decreased to between 0.2 and 0.3, indicating the uniformity of particle size in these formulations.

### 2.3. Transmission Electron Microscopy (TEM) Assay

**Figure 3:**
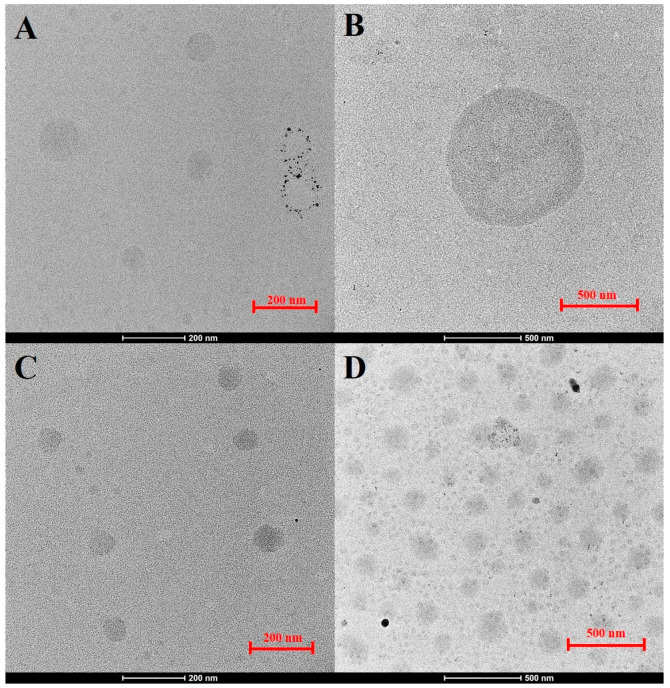
The effect of crude extract on the sizes of CO_2_-NBs in different formulations according to TEM analysis. (**A**) CO_2_-NBs alone, (**B**) CO_2_-NBs with crude extract, (**C**) CO_2_-NBs with Sp60, and (**D**) CO_2_-NBs with crude extract and Sp60.

TEM analysis illustrated notable variations in the size of CO_2_-NBs among four formulations on day 0: CO_2_-NBs alone, CO_2_-NBs + crude extract, CO_2_-NBs + Sp60, and a combination of CO_2_-NBs + crude extract + Sp60, as depicted in Figure 3. The formulation of CO_2_-NBs with crude extract exhibited relatively large nanobubbles with an average diameter of 1235 nm. In contrast, the inclusion of Sp60 in CO_2_-NBs with crude extract reduced the average diameter to 446 nm and resulted in a more uniform, well-dispersed structure.

### 2.4. Zeta Potential and pH Value

**Figure 4:**
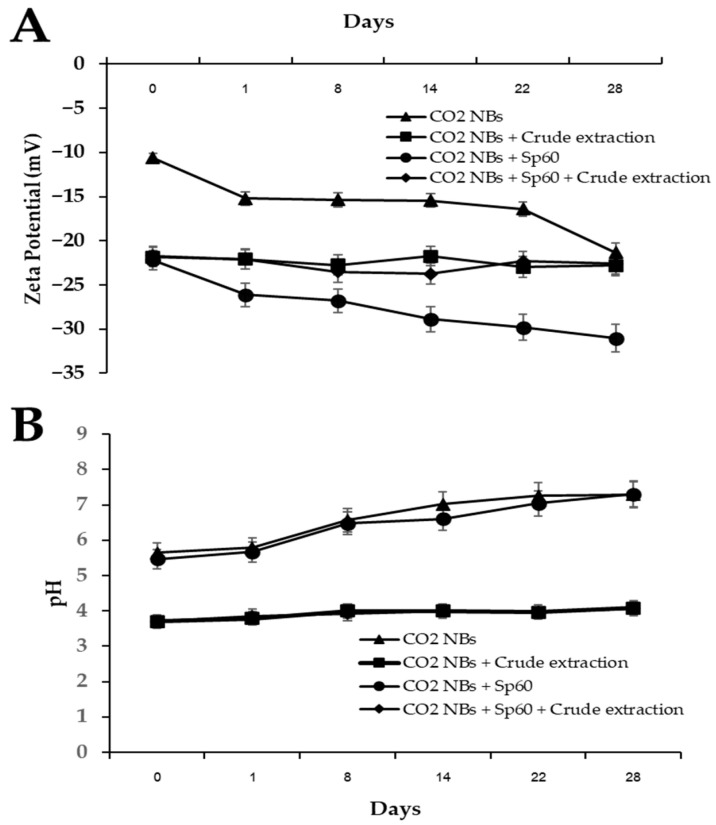
Zeta potential (**A**) and pH value (**B**) of CO_2_-NBs versus surfactant complexes.

It was found that the zeta potential value for days 0–28 varied, with each group showing different trends of change, as shown in Figure 4A. The zeta potential value of CO_2_-NBs was −10.63 mV on day 0 and decreased gradually over time, reaching −21.33 mV on day 28. The CO_2_ NBs + Sp60 treatment showed the lowest zeta potential (−31.03 mV) on day 28, which had a significant effect on particle stability. CO_2_-NBs + crude extract and CO_2_-NBs + Sp60 + crude extract tended to be relatively stable, with a zeta potential value of −21.73 to −22.80 mV.

The pH values of CO_2_-NBs and CO_2_-NBs + Sp60 remained stable, ranging from approximately 5.5 to 7.3. In contrast, CO_2_-NBs + crude extract and CO_2_-NBs + Sp60 + crude extract showed pH values in the range of 3.70–4.10, indicating an acidic environment attributed to the formation of carbonic acid (Figure 4B).

### 2.5. Minimum Inhibitory Concentration (MIC) and Minimum Bactericidal Concentration (MBC) Assay

The CO_2_-NBs with crude extract were evaluated in terms of their inhibitory activity against *X. oryzae*. CO_2_-NBs + crude extract and CO_2_-NBs + crude extract + Sp60 exhibited inhibitory activity against *X. oryzae,* with MIC and MBC values of 64 μg/mL and 128 μg/mL, respectively (Table 1).

### 2.6. Disease Suppression Assay

**Figure 5:**
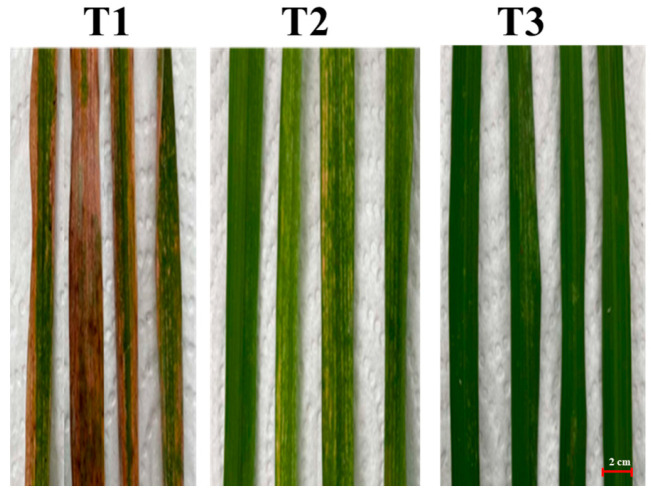
The effect of CO_2_ M-NB surfactant complexes coated with crude ethyl acetate extract on the control of bacterial leaf blight. (T1 = control, T2 = bioactive CO_2_-NBs, T3 = kanamycin (antibacterial)).

The reduction in disease severity was observed after fourteen days of *X. oryzae* infection (Figure 5). Symptoms of the initial leaf blight were observed in the control treatment after seven days of *X. oryzae* infection. The results reveal that when *X. oryzae* was treated with CO_2_-NBs + crude extract + Sp60 at a concentration of 128 μg/mL, the disease severity index (DSI) was 43%. This value is significantly lower than that of the control treatment inoculated with *X. oryzae* only, which had a DSI of 97%. Additionally, CO_2_-NBs + crude extract + Sp60 were able to reduce the severity of leaf blight caused by *X. oryzae* by 57%, as shown in Table 2.

## 3. Discussion

The present study demonstrated that the crude ethyl acetate extract of *T. polyalthiae* UB-ZSN2-1 exhibited strong antibacterial activity against *X. oryzae* pv. *oryzae*, consistent with previous reports describing the antimicrobial potential of violaceol-derived compounds produced by endophytic fungi [17]. The activity observed is consistent with the chemical nature of violaceol derivatives, whose conjugated aromatic frameworks and hydroxyl functionalities facilitate interactions with microbial membranes and key metabolic targets [18]. These structural attributes likely contribute to the disruption of essential cellular processes in *X. oryzae* pv. *oryzae,* thereby accounting for the potent inhibitory effect, efficacy comparable to conventional antibiotics [17,19]. Direction for future work that detailed chemical characterization (e.g., HPLC or LC–MS analysis) is required to confirm extract composition and ensure quality control.

The physicochemical properties of CO_2_-NBs observed in this study suggest a mechanistic interplay between surfactants, crude extracts, and bubble stability. The addition of the Sp60 surfactant reduced the nanobubble size to 140–259 nm, whereas the crude extract alone resulted in larger bubbles (1235–2972 nm). TEM and DLS analyses provide complementary size information, with DLS reflecting hydrodynamic diameter in suspension and TEM showing particle morphology under dry conditions. Treatments have lower PDI values exhibited more uniform and well-dispersed nanobubbles in TEM, indicating improved colloidal stability. Higher absolute zeta potential values were associated with reduced PDI and enhanced homogeneity [20]. This indicates that Sp60 functions as a steric and electrostatic stabilizer, adsorbing at the gas–liquid interface to reduce surface tension and prevent coalescence, producing smaller and more uniform nanobubbles [21]. Mechanistically, the crude fungal extract likely contributes macromolecular layers (proteins, polysaccharides, polyphenols) [22] that increase surface roughness and bubble aggregation. When combined with Sp60, the surfactant interacts with these biomolecules at the interface, creating a steric barrier that stabilizes the bubbles and enhances monodispersity (PDI 0.2–0.3). This synergistic effect suggests that surfactants and bioactive macromolecules collectively modulate interfacial energy and particle uniformity. This mechanistic interplay is consistent with prior studies showing that amphiphilic biomolecules modified nanobubble interfaces, altering size distribution and surface charge [23]. The enhanced monodispersity in the combined formulation indicates that surfactant-mediated stabilization mitigates the destabilizing effect of bioactive macromolecules, maintaining a stable colloidal system. This finding aligns with earlier reports that the combined use of surfactants and natural extracts can synergistically enhance nanobubble stabilization by reducing interfacial tension and providing steric hindrance [12,24].

Zeta potential dynamics provide further mechanistic insight. The CO_2_-NBs alone became more negatively charged over time (from −10.63 mV to −21.33 mV), likely due to hydroxyl ion accumulation and proton release at the gas–liquid interface. Sp60 increased the negative surface charge to −31.03 mV, stabilizing the colloidal system via electrostatic repulsion. This is caused by the interfacial accumulation of hydroxyl ions (OH^−^) and the preferential release of protons (H^+^) from the gas–liquid interface [25,26]. Resulting in their surface structure reorganizes, leading to more ordered interfacial water layers and increased negative surface charge density, resulting in improved colloidal stability [27,28,29]. Interestingly, crude extract-containing formulations maintained moderate zeta potentials (−21.73 to −22.80 mV), implying stabilization through hydrogen bonding and weak electrostatic interactions rather than strong surface charge effects. Previous studies have reported that biomolecules from microbial extracts can impart moderate stability through hydrogen bonding and weak electrostatic interactions, but are generally less efficient than synthetic surfactants in generating strong interfacial charges [30]. The acidic pH (3.70–4.10) in crude extract formulations can enhance antibacterial activity by promoting CO_2_ dissolution and carbonic acid formation at the interface, facilitating the release of bioactive metabolites [31]. The acidic microenvironment could have biological implications, particularly for antimicrobial applications, since low pH conditions can inhibit microbial growth and enhance the activity of bioactive compounds from fungal extracts [32]. The antibacterial assays revealed that CO_2_-NBs alone exhibited no significant activity (MIC > 128 μg/mL), whereas the combination with crude extract reduced MIC to 64 μg/mL and MBC to 128 μg/mL. This is consistent with previous studies reporting that *T. polyalthiae* UBZSN2-1 produced violaceol I and violaceol II, which have MIC values at <9.765–156.25 μg/mL of antimicrobial activity against human pathogens, including Gram-positive bacteria [17]. Kanamycin, used as a positive control against *X. oryzae*, has been reported to show MIC values typically ≥ 100 μg/mL due to partial resistance [33]. In comparison, the CO_2_-NBs with crude extract combination demonstrated comparable or slightly improved inhibitory activity. Although its antibacterial efficacy remains moderate relative to highly potent antibiotics, these findings suggest that the combined formulation has potential as an alternative or complementary antibacterial agent, warranting further optimization and evaluation. This suggests a delivery-focused mechanism: CO_2_-NBs enhance the dispersion and penetration of bioactive fungal metabolites into bacterial cells rather than acting as bactericidal agents themselves [34]. Encapsulation within the nanobubble interface may balance delivery and sequestration of bioactives, consistent with surfactant-mediated carrier systems in other studies [20,35]. The disease suppression results in a reduction in bacterial leaf blight severity by 57% under greenhouse conditions. These results indicate that nanobubble-mediated delivery improves foliar uptake and local concentration of bioactive compounds on rice leaves. By increasing the contact surface area and penetration into leaf tissues [36,37], CO_2_-NBs act as carriers that potentiate the antifungal effect of crude extract [38], aligning with prior reports of gas nanobubbles enhancing nutrient and antimicrobial delivery in plants [39]. Although CO_2_-NBs combined with crude extract and Sp60 reduced disease severity by 57%, this was still lower than the 80% reduction achieved by chemical bactericide. This partial suppression may be due to the crude extract containing mixed metabolites with lower potency and specificity than synthetic agents [40]. Additionally, although nanobubbles improve dispersion, bioactive compounds may still undergo degradation or show limited penetration and distribution on leaf surfaces under greenhouse conditions [41]. Chemical bactericides typically act through direct, highly efficient mechanisms, whereas fungal metabolites often rely on slower or multi-pathway effects [42]. Improving nanobubble adhesion, metabolite loading, or formulation ratios may enhance performance and help close the gap with commercial treatments.

The findings indicate that combining CO_2_-NBs with bioactive extracts offers a promising alternative to chemical bactericides. Nanobubble-mediated delivery could reduce the required dose of bioactive compounds, minimize environmental impact, and decrease the risk of resistance development. Optimizing formulation parameters, such as surfactant concentration and extract-to-nanobubble ratios, may further enhance efficacy. Future studies should explore molecular-level interactions between nanobubbles, plant surfaces, and pathogens, as well as field-scale validation under diverse agroecological conditions.

## 4. Materials and Methods

### 4.1. T. polyalthiae UBZSN2-1 Preparation

The endophytic fungal strain used in this study, *T. polyalthiae* UBZSN2-1, isolated from the *Polyalthia debilis* which previously reported as a new species by Nuankeaw et al. [16]. It produces violaceol I and violaceol II, which can inhibit bacteria [17]. Violaceol I and violaceol II are phenolic secondary metabolites belonging to the bisnaphthopyrone group. Their structures contain conjugated aromatic rings and hydroxyl groups, which influence both their biological activity and extraction behavior. Because these compounds exhibit moderate polarity, organic solvents such as ethyl acetate or methanol are typically effective for their extraction from fungal cultures. The fungus was obtained from the Mycorrhiza and Mycotechnology Laboratory, Department of Microbiology, Faculty of Science, Khon Kaen University, Thailand. Fungal endophyte culture was inoculated onto PDA (HiMedia™, Mumbai, India) and then incubated at 30 °C for 7 days. *T. polyalthiae* UBZSN2-1 was then used as an inoculum in the next experiment.

### 4.2. Extraction and Screening of Bioactive Compounds from T. polyalthiae UBZSN2-1

The endophytic fungus was cultured on potato dextrose agar (PDA; HiMedia™, India) at 30 °C for 7 days. After that, 200 mycelial plugs (0.5 cm; cork borer no.5) were inoculated into 5000 mL Erlenmeyer flasks, each containing 2000 mL of sterilized (using an autoclave at a temperature of 121 °C and a pressure of 15 pounds per square inch for 150 min) potato dextrose broth (PDB; HiMedia™, India), and incubated at room temperature for 30 days under static conditions. The bioactive compounds in each culture broth were extracted by filtering through cotton prior to extraction with ethyl acetate (EtOAc) in a ratio of 1:10 (*v*/*v*) and then concentrated via rotary evaporator to obtain crude EtOAc extracts, which were screened for antibacterial activity using the disc diffusion method [5]. Briefly, sterile paper discs (6 mm in diameter) were impregnated with the crude ethyl acetate extract dissolved in 10% DMSO at 128 µg/mL and placed on the nutrient agar (NA) plates previously inoculated with the test microorganism. Chloramphenicol was used as a positive control. After incubation at 37 °C for 24 h, the inhibition zone around each disc was measured to evaluate the antibacterial efficacy.

### 4.3. Preparation of CO_2_-NB/Bioactive Surfactant Complexes

#### 4.3.1. Generation of CO_2_-NBs

**Figure 6:**
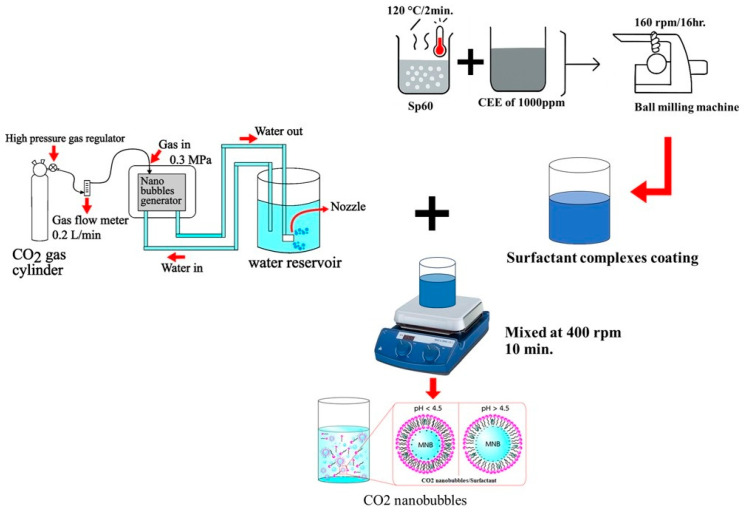
A schematic representation of the preparation steps for CO_2_ micro-nanobubble surfactant complexes coated with bioactive compounds.

CO_2_ nanobubbles (CO_2_-NBs) were generated using the decompression method described by Traiphop et al. [10]. The system consisted of a stainless-steel pressurized vessel (capacity 10 L) equipped with a centrifugal pump and a CO_2_ gas inlet line. Deionized water and CO_2_ gas (purity ≥ 99.9%; KASIDITH TRADING Co., Ltd., Khon Kaen, Thailand) were introduced into the vessel and circulated at flow rates of 10 L/min and 0.2 L/min, respectively, under a hydraulic pressure of 0.3 MPa for 10 min. The high-pressure CO_2_-saturated water was then rapidly depressurized through a release valve to generate nanobubbles, as shown in Figure 6. The CO_2_-NB solution was then allowed to rest in a plastic bottle at room temperature for 3 days to reach equilibrium and stabilize, allowing for any immediate reactions or changes in bubble size and distribution.

#### 4.3.2. Preparation of Crude Extract and Surfactant Solutions

The crude ethyl acetate extract (CEE) dissolved in 10% DMSO was incorporated into reverse osmosis (RO) water to obtain a 1000 mg/mL concentration. The surfactant used to encapsulate the CO_2_-NBs, sorbitan monostearate (Sp60, Krungthepchemi Co., Ltd., Bangkok, Thailand), was prepared using ball milling techniques [43]. Initially, Sp60 was heated to approximately 120 °C for 2 min, then dissolved in 20 mL of CEE solution and stirred for 5 min. This solution was subsequently transferred to 50 mL high-density polyethylene (HDPE) containers filled with 2 mm diameter spherical zirconia beads, occupying 3/4 of the container volume. Milling was carried out using a horizontal planetary ball milling machine at a rotational speed of 160 rpm for 16 h at room temperature (Figure 6).

#### 4.3.3. Formulation of CO_2_-NB/Bioactive Surfactant Complexes

The bioactive surfactant solution was prepared at 2.61 × 10^−5^ Molar. CO_2_-NBs were mixed with bioactive surfactants via self-assembly using a magnetic bar at 400 rpm for 10 min at room temperature. Subsequently, CO_2_-NBs complexed with bioactive surfactants were successfully produced. All samples were then maintained at room temperature for physical characterization.

### 4.4. Particle Size, Zeta Potential, and pH Measurements

The polydispersity index (PDI) was determined using dynamic light scattering (DLS) analysis (Malvern Zetasizer Nano ZS, Malvern Instruments, UK). The PDI value was automatically calculated using the Zetasizer software version v8.02 based on cumulant analysis of the intensity autocorrelation function, representing the width of particle size distribution. The pH value was measured with a pH meter (PH-2 Pro Meter pH Litmus tester). For all measurements, three replications were performed for each formulation at room temperature.

### 4.5. Transmission Electron Microscopy (TEM) Assay

The morphology and structure of the samples were analyzed using a Transmission Electron Microscope (TEM, Talos F200X G2, Thermo Fisher Scientific Inc., Waltham, MA, USA). The samples were prepared by drop-casting a diluted sample onto a carbon-coated copper grid and allowing it to dry at room temperature. TEM images were captured at an accelerating voltage of 120 kV.

### 4.6. Minimum Inhibitory Concentration (MIC) and Minimum Bactericidal Concentration (MBC) Assays

The MIC assay was performed using a two-fold serial dilution method in a microdilution plate. *Xanthomonas oryzae* pv. *oryzae* was inoculated in peptone yeast glycerol (PYG) medium and incubated at 37 °C for 24 h. The crude ethyl acetate extract, dissolved in 10% DMSO, and CO_2_-NB/bioactive surfactant complexes were incorporated into the PYG medium to obtain a concentration of 256 μg/mL, which was then serially diluted to 128, 64, 32, 16, 8, 4, 2, 1, 0.5, and 0.25 μg/mL. Bacterial cell turbidity was adjusted to 0.08–0.10 (10^6^ CFU/mL) using a spectrophotometer (Hitachi High-Tech Corporation, Tokyo, Japan) at 600 nm. Kanamycin was used as the positive control. The microdilution plate was incubated at 37 °C for 24 h. After incubation, 10 μL of 0.18% resazurin solution was added to all wells. The lowest concentration at which the resazurin color did not change to pink was interpreted as the MIC, expressed in μg/mL, which represents the lowest concentration that inhibited the growth of *X. oryzae* pv. *oryzae*. Furthermore, three concentrations—below the MIC, at the MIC, and above the MIC—were streaked on the PYG medium to determine the minimum bactericidal concentration (MBC). The MBC was defined as the lowest concentration at which no growth of *X. oryzae* pv. *oryzae* was observed.

### 4.7. Disease Suppression Assay in Rice

#### 4.7.1. Experimental Design and Rice Cultivation

The pot experiments were set up at the Agronomy Farm, Khon Kaen University, Thailand (16°28′ N, 102°48′ E, 200 m above mean sea level). A completely randomized design (CRD) with three treatments and nine replications was employed. The rice plants were cultivated in pots (12 inches in diameter) under greenhouse conditions. The climate consists of an average temperature of 18–30 °C, 70–80% moisture, 12 h of sunlight, and a wind speed of 11.2 km/h. Three treatments were carried out as follows:

T1: plants treated with *X. oryzae* (control).
T2: plants treated with *X. oryzae* and bioactive CO_2_-NBs.
T3: plants treated with *X. oryzae* and kanamycin (antimicrobial).

The chemical and physical properties of soil samples were determined prior to cultivation. The chemical compositions and physiological properties of the soil were as follows: sandy loam soil with a pH 5.9, electrical conductivity (EC) of 0.14 dS/m, organic matter (OM) content of 0.640%, nitrogen (N) content of 0.060 mg kg^−1^, phosphorus (P) content of 145.29 mg kg^−1^, potassium (K) content of 88.01 mg kg^−1^, available phosphorus content of 22.70 mg kg^−1^, exchangeable potassium content of 50.20 mg kg^−1^, calcium (Ca) content of 790.26 mg kg^−1^, sodium (Na) content of 29.17 mg kg^−1^, and magnesium (Mg) content of 67.97 mg kg^−1^.

Black upland rice (*Oryza sativa* subsp. *indica*) was provided by the group conducting the Rice project, Faculty of Agriculture, Khon Kaen University, Thailand. Rice seeds were surface-sterilized via soaking in a 6% sodium hypochlorite solution for 5 min. Sterilized rice seeds were transferred to trays containing sterilized peat moss. The rice plants were grown for 2 weeks before transplantation to pots (12 inches in diameter).

#### 4.7.2. Biological Control of Bacterial Leaf Blight Caused by *Oryza sativa* L. Using Bioactive CO_2_-NBs Under Greenhouse Conditions

A pot culture experiment was conducted to test the efficiency of bioactive CO_2_-NBs against *X. oryzae* under greenhouse conditions. After 45 days of transplantation, rice plants were first treated with bioactive CO_2_-NBs at a concentration of 128 µg/mL and distilled water was used as the control. The treatment was allowed to remain on the plants for 2 days to ensure sufficient absorption and interaction with plant tissues before pathogen challenge. Following this waiting period, *X. oryzae* cultured in NB medium at 150 rpm and 28 °C for 24 h was prepared as a suspension at 4.5 × 10^6^ CFU/mL and sprayed onto the leaves. Disease development was monitored for 15 days after inoculation, or when complete symptoms of the disease were fully expressed. Lesion length was recorded, and then the disease severity index and the percentage of disease reduction for each treatment were calculated [44].

### 4.8. Data Collection

Plants with bacterial leaf blight (defined as all plant leaves showing bacterial leaf blight symptoms) were considered to present disease incidence according to the evaluation standards of the International Rice Research Institute (IRRI) [45], which classifies disease severity based on the percentage of leaf area affected. The period from the first day of incubation until permanent wilting appeared was recorded to determine disease severity. The disease severity rating scales used to score individual plants are shown in Table 3. These scores were used to calculate the disease severity index and disease reduction for each treatment [46].

### 4.9. Statistical Analysis

Data were analyzed using the Statistix 10 software. This step involved an analysis of variance (ANOVA) for data in CRD. Fisher’s Least Significant Difference (LSD) test was used to determine 95% confidence intervals (*p* ≤ 0.05).

## 5. Conclusions

In summary, the application of CO_2_ nanobubbles (CO_2_-NBs) in combination with a crude extract of *T. polyalthiae* UBZSN2-1 reduced leaf blight disease in rice by more than 50% under greenhouse conditions. This study demonstrates the efficacy of using new delivery formats with bioactive compounds as an alternative to control plant diseases. These findings pave the way for the development of bioactive CO_2_-NBs as replacements for biological compounds or synthetic chemicals in disease control and plant cultivation. This approach supports the establishment of more sustainable rice production systems, offering farmers an environmentally friendly and sustainable alternative for rice cultivation. Nevertheless, future studies should include comparative experiments with other gases (e.g., SO_2_ and NH_3_) to gain a deeper mechanistic understanding of gas-specific effects on aqueous pH stability and nanobubble behavior.

## Figures and Tables

**Table 1:** Effect of CO_2_-NBs plus crude extract on *X. oryzae*.

Treatment	MIC (μg/mL)	MBC (μg/mL)
Crude EtOAc extract	128	-
CO_2_-NBs	>128	>128
CO_2_-NBs with crude extract	64	128
CO_2_-NBs with Sp60	>128	>128
CO_2_-NBs with crude extract and Sp60	64	128

**Table 2:** Effects of CO_2_-NBs and crude extract on growth inhibition of leaf blight disease in rice plants infected by *X. oryzae* under greenhouse conditions.

Treatment	Disease Severity Index (%)	Disease Reduction (%)
No inoculation with the pathogen	0.00 ± 0.00	0.00 ± 0.00
*X. oryzae*	97.00 ± 0.18 ^a^	0.00 ± 0.00 ^c^
CO_2_-NBs + crude extract + Sp60 + *X. oryzae*	43.00 ± 0.12 ^b^	57.00 ± 0.13 ^b^
Bactericide + *X. oryzae*	20.00 ± 0.08 ^c^	80.00 ± 0.09 ^a^
% CV	19.30	21.14
F-Test	**	**

Numbers followed by the different superscript letters in each column indicate that the data are significantly different according to the LSD test. ** Significant difference at *p* ≤ 0.01.

**Table 3:** Disease score ratings for bacterial leaf blight in greenhouse test [45].

Lesion Length (cm)	Score	Description
1–5	1	resistant (R)
>5–10	2	moderately resistant (MR)
>10–15	3	moderately susceptible (MS)
>15–20	4	susceptible (S)
>20	5	highly susceptible (HS)

## Data Availability

The datasets obtained and analyzed in the current study are available from the corresponding author upon reasonable request.
